# Im Spannungsfeld zwischen Sicherheit und Freiheit: Eine Analyse zur Akzeptanz der Corona-Warn-App

**DOI:** 10.1365/s40702-020-00646-3

**Published:** 2020-08-06

**Authors:** Wassili Lasarov

**Affiliations:** Professur für Marketing, Christian-Albrechts-Universität zu Kiel, Westring 425, 24118 Kiel, Deutschland

**Keywords:** Corona, Corona-Warn-App, Datenschutz, Datensicherheit, Privatsphäre, Coronavirus, Corona-warning-app, Data protection, Data security, Privacy

## Abstract

Um Infektionsketten in der Corona-Pandemie effektiv nachzuverfolgen und zu unterbrechen, wurde die Corona-Warn-App in Deutschland eingeführt. Diese sogenannte Tracing-App verfolgt Begegnungen zwischen den Anwendern der App und warnt sie, falls sie mit Infizierten in Kontakt getreten sind. In der Öffentlichkeit entbrannte im Zuge der Einführung der Corona-Warn-App eine intensive Diskussion über den Umgang mit persönlichen Daten bei der Verwendung der App (z. B. durch die vermeintliche Erfassung der Standortdaten) sowie über die Wirksamkeit der App (z. B. durch mangelnde Teilnahme, insbesondere von älteren Menschen). Die vorliegende Studie untersucht, welche Einflussfaktoren die Akzeptanz der Corona-Warn-App erhöhen oder mindern können. Es werden hierfür relevante Einflussfaktoren aus der bisherigen Literatur ermittelt und auf den Anwendungsfall der Corona-Warn-App übertragen. Mit diesen Faktoren wurde ein Kategorienschema entwickelt, auf dessen Basis in einer qualitativen Studie 967 Leserkommentare mittels Inhaltsanalyse untersucht wurden. Die Ergebnisse bestätigen, dass besonders drei Kategorien relevant in diesem Zusammenhang sind: Der Nutzen der Corona-Warn-App (37 % der Nennungen), Datenschutz, Privatsphäre, Transparenz und Vertrauen (26 %) sowie der Einfluss der sozialen Umwelt (8 %). Zum Schluss werden Implikationen für zukünftige Forschung kurz vorgestellt.

## Abstract (en)

One of the major challenges in slowing the spread of the coronavirus is that covid-19 may be contagious before symptoms are noticeable. For this reason, the German government launched a corona tracing app (“Corona-Warn-App”). Contact tracing for COVID-19 requires identifying people who may have been exposed to COVID-19 and following them up daily for 14 days from the last point of exposure. Therefore, the app warns users if they might have been in close contact with COVID-19-infected individuals. When systematically applied, contact tracing will break the chains of transmission of COVID-19 and is an essential public health tool for controlling the virus. The German corona tracing app has been discussed in public, particularly as many people have strong privacy concerns. Furthermore, many people questioned the effectiveness of the tracing-app (e.g., as many older people are not willing or capable of using the app). The present study sheds light on current research on individual privacy concerns in light of the launch of the corona tracing app. Building on this, the author presents psychological determinants of individual privacy concerns that have been discussed in research so far. The author analyses in a qualitative study 967 reader comments to determine whether the previously identified individual determinants are relevant in the case of the Corona-Warn-App. The results confirm that three categories in particular are relevant in this context: The functional utility of the app (37% of codings), data protection, privacy, transparency and trust (26%) and the influence of the social environment (8%). Finally, implications for further research are derived.

## Einleitung

Eine große Herausforderung bei der Eindämmung der Corona-Pandemie ist, dass das Coronavirus (SARS-CoV-2/Covid-19) schon ansteckend sein kann, bevor Infizierte Symptome entwickeln (RKI 2020). Somit kann es in einigen Fällen schwer nachzuvollziehen sein, wann und wo sich eine bestimmte Person infiziert hat und wen sie angesteckt haben könnte. Daher sollten Personen, die mit Infizierten in Kontakt standen, frühzeitig gewarnt werden, um sogenannte Infektionsketten zu unterbrechen. Aus diesem Grund wurde am 16.06.2020 die *Corona-Warn-App* in Deutschland eingeführt (Bundesregierung.de 2020). Die App, deren gesamter Quellcode Open Source ist (Github.com 2020), wurde im Auftrag des Robert-Koch-Instituts programmiert und soll dabei helfen, auch nach der Lockerung der Einschränkungen des öffentlichen Lebens die Ausbreitung des Coronavirus zu vermeiden. Mithilfe dieser sogenannten *Tracing-App*^1^ werden Begegnungen zwischen Nutzern der App verfolgt. Dadurch können diese effektiver gewarnt werden, falls sie mit Infizierten in Kontakt getreten sind. Die Benutzung der Corona-Warn-App soll den Zeitraum vom positiven Testergebnis einer infizierten Person bis zur Benachrichtigung von Kontaktpersonen verkürzen. Damit soll die Kontaktnachverfolgung schneller und zuverlässiger erfolgen als bei einer händischen Erfassung von Infizierten in Gesundheitsämtern, da sich positiv getestete Personen sonst daran erinnern müssen, welche Orte sie in den vergangenen 14 Tagen aufsuchten. Bereits eine Woche nach Einführung der App hatten ca. 12 Mio. Menschen die App geladen (Tagesschau.de 2020a). Trotz der hohen Downloadzahlen wurde die Corona-Warn-App insbesondere im Hinblick auf den Umgang mit persönlichen Daten (z. B. durch die vermeintliche Erfassung der Standortdaten) und die Wirksamkeit einer solchen App (z. B. durch mangelnde Teilnahme, insbesondere von älteren Menschen) kritisiert. So gaben bspw. 32 % der Probanden einer Umfrage an, für sie spräche gegen die Installation einer App zur Kontaktverfolgung vor allem die Sorge vor „mehr Überwachung nach der Epidemie“ (Abeler et al. 2020). Ein weiterer Kritikpunkt war, dass lediglich ca. 80 % der Bevölkerung überhaupt ein Smartphone verwenden (Bitkom 2020) und die App auf vielen älteren Geräten nicht funktioniert (Handelsblatt.com 2020a). Der Anteil der Smartphone-Nutzer ist zudem in jenen Altersgruppen geringer, die nach Angaben des Robert-Koch-Instituts als Risikogruppen gelten (RKI.de 2020; Zeit Online 2020a). Darüber hinaus lassen sich einige paradoxe Erscheinungen im öffentlichen Diskurs beobachten: So äußern viele Menschen ihre Bedenken hinsichtlich des Datenschutzes der Corona-Warn-App auf Social Media Plattformen wie bspw. Facebook, die ihrerseits häufig selbst in der Kritik wegen ihres Umgangs mit persönlichen Daten standen.

Aktuelle verhaltens- und sozialwissenschaftliche Forschung zum Umgang von Individuen mit persönlichen Daten befasst sich u. a. mit psychologischen Faktoren, die individuelle Datenschutzbedenken fördern oder hemmen (z. B. Aguirre et al. 2015). So untersuchen Studien besonders den individuellen Zielkonflikt, der aus zwei konkurrierenden Bedürfnissen in der digitalen Welt entsteht: Einerseits dem Bedürfnis nach einer möglichst effektiven Nutzung digitaler Technologien, um den individuellen Nutzen zu steigern (z. B. durch Personalisierung von Produktempfehlungen). Andererseits das Bedürfnis, die eigene Privatsphäre möglichst gut zu schützen und damit möglichst wenig Daten preiszugeben (Aguirre et al. 2015). Die vorliegende Studie überträgt diese Erkenntnisse auf den aktuellen gesellschaftlichen Diskurs über den Umgang mit persönlichen Daten im Zuge der Einführung der Corona-Warn-App. Dafür werden zunächst aus der bisherigen Literatur relevante psychologische Faktoren zum Umgang mit Datenschutzbedenken vorgestellt und im Lichte der aktuellen Diskussion um die Corona-Warn-App diskutiert. Abschließend werden in einer qualitativen Studie 967 Leserkommentare mittels Inhaltsanalyse dahingehend untersucht, ob diese Faktoren für die Steigerung oder Senkung der Akzeptanz der Corona-Warn-App tatsächlich relevant sein könnten oder ob sie um weitere psychologische Determinanten ergänzt werden müssen. Auf Basis dieser Studie sollte sich zukünftige Forschung in weiteren empirischen Untersuchungen (z. B. großzahlige quantitative Befragungen) den Besonderheiten des Falls widmen, um bestehende Theorien und Konzepte ggf. weiterzuentwickeln.

## Der untersuchte Fall: Die Corona-Warn-App

Die Corona-Warn-App erfasst mittels der Bluetooth-Technologie die Begegnungen zwischen zwei Personen, die sich die App auf ihren Smartphones installiert haben, ohne den genauen Standort der einzelnen Personen zu verfolgen. Dazu funkt die App in regelmäßigen Abständen (2,5–5 min) eine anonymisierte Identifikationsnummer 16 Mal in die nähere Umgebung. Zugleich prüft das Smartphone, ob es Bluetooth-Signale von anderen Geräten empfangen kann. Ist die App auf zwei Geräten installiert, die sich über einen längeren Zeitraum sehr nahekommen, tauschen diese temporäre Identifikationsnummern aus. Wird eine Person positiv auf das Coronavirus getestet, kann sie das in der App vermerken und zustimmen, dass alle ihre Kontaktpersonen gewarnt werden sollen. In diesem Fall werden die Tagesschlüssel der infizierten Person auf einen zentralen Server hochgeladen. Die Smartphones der App-Nutzer laden regelmäßig anonymisierte Listen mit den Tagesschlüsseln aller positiv Getesteten auf ihre Smartphones und können anhand der Listen lokal abgleichen, ob sich daraus temporäre Identifikationsnummern ableiten lassen, mit denen das Gerät Kontakt hatte. Gab es einen Kontakt, den die App als infektionsrelevant einstuft, erscheint eine Warnmeldung. Die Nutzer erhalten darüber hinaus einen Hinweis, dass sie getestet werden sollten (Bundesregierung.de/corona-warn-app.de).

## Aktuelle Forschung und Anwendung auf den Fall der Corona-Warn-App

In der heutigen Welt sind Individuen in vielen Lebensbereichen ständig von digitalen Technologien umgeben. Um die unzähligen digitalen Produkte und Dienstleistungen möglichst effektiv zu nutzen, ist zumeist die Bereitstellung persönlicher Daten notwendig, auf deren Basis individualisierte Angebote erstellt werden. So verarbeiten Video-on-Demand Dienstleister bisherige Film- und Serienvorlieben der Nutzer, um daraus persönliche Empfehlungen abzuleiten (z. B. Netflix). Online-Händler empfehlen personalisiert Produkte auf Basis früherer Käufe (z. B. Amazon), Geotracking Systeme navigieren ihre Nutzer unter Verarbeitung ihrer individuellen Standortdaten durch die Stadt (z. B. Google Maps), smarte Fitnessgeräte geben den Nutzern Rückmeldungen über ihre Schlafgewohnheiten (z. B. Fitbit), etc. Angesichts der automatisierten Verarbeitung der daraus entstehenden riesigen Datenmengen wird der verantwortungsvolle Umgang mit sensiblen und persönlichen Daten von Einzelpersonen, Unternehmen und öffentlichen Institutionen eines der wichtigsten Themen der Zukunft sein (Acquisti et al. 2015). Zwar bieten all die genannten Anwendungen erhebliche Vorteile für den Lebens- und Konsumalltag, allerdings kann dadurch auch die Privatsphäre von Verbrauchen ausgehöhlt werden und deren Autonomie als Konsumenten und Bürger bedrohen (Cohen 1999; Crawford et al. 2014). Neben staatlichen Regulationen (John et al. 2011) und der zunehmenden Selbstverpflichtung von Unternehmen zum verantwortungsvollen Umgang mit den Daten ihrer Kunden (Lobschat et al. 2019), suchen viele Menschen zunehmend nach individuellen Lösungen, um den Missbrauch ihrer persönlichen Daten zu vermeiden. So melden sich bspw. viele Nutzer von sozialen Medien ab (Edelman 2018) oder geben absichtlich falsche Daten weiter (DePaulo et al. 2003).

In der bisherigen Literatur existieren einige konzeptionelle Ansätze, die psychologische Einflussfaktoren im Zusammenhang mit Datenschutzbedenken und der Akzeptanz von Technologien untersuchen. So beschäftigten sich beispielsweise Martin und Murphy (2017) in einem konzeptionellen Rahmen mit Datenschutzbedenken im Marketingkontext. Acquisti et al. (2015a) verknüpfen verschiedene Ströme empirischer Forschung zum Datenschutzverhalten in einer konzeptionellen Studie miteinander. Darüber hinaus trifft das *Technology Acceptance Model* (TAM) sowie dessen Weiterentwicklungen (z. B. TAM2) Aussagen darüber, warum Individuen Technologien nutzen (Schepers 2007). Im Folgenden werden aus diesen Rahmenmodellen und Theorien drei ausgewählte Themenfelder diskutiert, die für den Kontext der Corona-Warn-App besonders relevant sind. So kann zunächst ein hoher individueller und gesellschaftlicher Nutzen einer Technologie einen persönlichen Zielkonflikt auslösen, in dem Individuen zwischen den Vorteilen für sich und die Gesellschaft und dem Schutz der eigenen Privatsphäre abwägen. Weiterhin ist die Beurteilung über die Verwendung und Preisgabe der eigenen persönlichen Daten facettenreich und kann bei vielen Menschen ein hohes Maß an Unsicherheit und Misstrauen gegenüber Unternehmen oder Institutionen auslösen. Um dieser Unsicherheit entgegenzutreten, kann die soziale Umwelt als Orientierung dienen, von der man „richtiges“ Handeln für sich ableitet. Gleichzeitig kann der Einfluss der sozialen Umwelt Individuen unter Druck setzen, sich konform zu sozialen Erwartungen zu verhalten.

### Der Zielkonflikt zwischen dem Nutzen der Technologien und dem Schutz der Privatsphäre

Individuen sehen sich im digitalen Zeitalter ständig einem Zielkonflikt gegenüber: Einerseits sollen die Möglichkeiten digitaler Technologien vollumfänglich ausgeschöpft werden, was meist nur über die Preisgabe persönlicher Daten möglich ist. Andererseits soll die eigene Privatsphäre möglichst gut geschützt werden, was wiederum durch die Zurückhaltung persönlicher Daten ermöglicht wird (z. B. Aguirre et al. 2015b). Interessanterweise geben zwar viele Menschen an, dass sie sehr besorgt um ihre Daten sind, allerdings weicht ihr Verhalten in konkreten Situationen oft von diesen geäußerten Einstellungen ab (Smith et al. 1996; Acquisti et al. 2015a). Diese individuelle Einstellungs-Verhaltens-Diskrepanz wird als *Privacy-Paradox* bezeichnet (Acquisti et al. 2015a). Eine Ursache hierfür ist, dass sich abstrakte Einstellungen selten in konkrete Verhaltensweisen übersetzen lassen und diese oftmals sogar stark voneinander abweichen können (Acquisti et al. 2015a). Eine weitere Ursache für diese Diskrepanz ist, dass Konsumenten ihre persönlichen Kosten, also die Freigabe ihrer persönlichen Daten, nicht immer wirklich einschätzen können und der beschriebene Zielkonflikt ihnen in konkreten Situationen gar nicht präsent ist (Acquisti et al. 2015a). So sind sich Menschen oft unsicher, ob sie überhaupt besorgt über die Verwendung ihrer persönlichen Daten sein sollten (Acquisti et al. 2015a). Ferner sind Verbraucher häufig bereit, höhere nicht-monetäre Kosten (z. B. die Weitergabe persönlicher Daten) auf sich zu nehmen, um sehr nützliche Produkte zu konsumieren (Monroe 2003). Eine empirische Studie zeigt, dass Menschen mit weniger Sorge um ihre offengelegten persönlichen Daten reagieren, wenn sie das Produkt als nützlich erachten (White et al. 2008). Ein und dieselbe Person kann sich also, einer einfachen Kosten-Nutzen-Kalkulation folgend, in verschiedenen Situationen unterschiedlich besorgt über die Verwendung der eigenen Daten zeigen bzw. mit dieser Besorgtheit unterschiedlich umgehen.

Der diskutierte Zielkonflikt zwischen dem persönlichen Nutzen und der der Offenlegung persönlicher Daten wurde auch mit der Einführung der Corona-Warn-App sichtbar. Abweichend von diesem Prinzip ist lediglich, dass sich der Konflikt diesmal eher zwischen persönlicher Freiheit und Sicherheit ergibt. So könnte eine zentralisierte und verpflichtende App das Leben während der Corona Pandemie sicherer machen, ginge aber zulasten der individuellen Freiheit. Eine Studie von epidemiologischen Modellierern der Oxford University bestätigte, dass die Nutzung einer Tracing-App positive Effekte in der Eindämmung der Corona-Pandemie haben könne (Ferretti et al. 2020). Das Virus verbreite sich laut ihren Modellrechnungen zu schnell, um über manuelle Kontaktverfolgung gestoppt zu werden. Mittels digitaler Kontaktverfolgung könne die Gesellschaft allerdings epidemische Kontrolle erlangen. Dies gilt allerdings nur, wenn die App von genügend Menschen genutzt wird.

### Unsicherheit und Vertrauen im Hinblick auf die Nutzung persönlicher Daten

Wie bereits erwähnt, sind sich Menschen oft unsicher, ob und in welchem Ausmaß sie überhaupt besorgt über die Verwendung ihrer persönlichen Daten sein sollten (Acquisti et al. 2015a). Diese Unsicherheit entsteht auch daraus, dass die Verbraucher den Umfang der gesammelten Daten oftmals nicht beurteilen können. Während manche Dienste die manuelle Eingabe von vielen persönlichen Daten erfordern (z. B. Angabe von Namen und Geburtsdatum), erfassen andere Dienste nach einmaliger Freigabe durch die Nutzer weitere Daten im Hintergrund (z. B. Geotracking-Systeme). Für viele Technologien reichen wiederum anonymisierte (oder wenigstens pseudonymisierte) Nutzerdaten, die auf aggregierter Ebene ausgewertet werden und bei denen Rückschlüsse auf einzelne Personen gar nicht oder nur sehr schwer möglich sind. Digitale Technologien unterscheiden sich folglich nicht nur hinsichtlich der geforderten Fülle an Daten, sondern auch hinsichtlich des Bewusstseins der Anwender über die Datenweitergabe. Darüber hinaus existieren oftmals Informationsasymmetrien zwischen Anbietern und Nutzern. So wird bspw. die Technologie, die hinter der Sammlung und Bearbeitung von persönlichen Daten steht, für Menschen ohne fachlichen Hintergrund immer schwerer nachvollziehbar (Acquisti et al. 2015a). Aus dieser Unwissenheit heraus kann eine sehr große Verunsicherung seitens der Verbraucher entstehen, die sich wiederum in starken Datenschutzbedenken ausdrücken kann. Ein Faktor, der dieses Gefühl reduziert und nicht unmittelbar mit dem Umgang mit persönlichen Daten zusammenhängt, ist das allgemeine Vertrauen von Individuen in die Institutionen und Unternehmen, denen sie ihre Daten zur Verfügung stellen (Ferdinand et al. 2020). So konnten Studien zeigen, dass Vertrauen die Bereitschaft von Konsumenten erhöht, ihre Daten freizugeben, die Produkte des Unternehmens zu kaufen oder dessen Dienstleistungen zu nutzen (Aiken und Boush 2006; Bart et al. 2005; Schlosser et al. 2006). Allerdings ist dieses Vertrauen äußerst fragil und kann bei Verletzung das Verhältnis von Individuen zu Unternehmen oder Institutionen nachhaltig beeinträchtigen (z. B. Miyazaki 2008).

Angesichts der umfangreichen Berichterstattung und des gesellschaftlichen Diskurses über potentielle Verletzungen der Privatsphäre durch eine Corona-Warn-App, wurden bereits im Vorfeld die Datenschutzaspekte der App von vielen Seiten intensiv beleuchtet (Zeit Online 2020b). So wurden 10 Prüfsteine vom Chaos Computer Club definiert, an denen sich die Entwickler der App orientieren sollten (CCC 2020). Den gesellschaftlichen Datenschutzbedenken folgend, konnte die fertiggestellte Tracing-App schließlich weder auf GPS-Daten zugreifen, noch über andere Wege den Aufenthaltsort der Nutzer ermitteln, was als wichtige Bedingung für eine möglichst hohe Verbreitung der App angesehen wurde (Zeit Online 2020c). Es wurden zudem keine persönlichen Daten bei der Anmeldung in der App abgefragt. Die Corona-Warn-App basierte auf einem dezentralen Ansatz der Datenspeicherung, d. h. die Begegnung zwischen Nutzern wurde auf jedem einzelnen Gerät gespeichert und nicht zentral hinterlegt (z. B. auf Servern). Da die temporären Identifikationsnummern alle 15 min aus einem Tagesschlüssel generiert wurden und ständig wechselten, waren auch darüber keine direkten Rückschlüsse auf die Nutzer möglich. Überdies war die App vor Missbrauch durch die Anwender geschützt (z. B. Falschmeldungen), da Infizierte ihr Testergebnis beim Eintragen in die App belegen mussten (z. B. durch einen QR Code vom Gesundheitsamt). Ferner war der Quellcode der App seit ihrer Einführung öffentlich verfügbar (Zeit Online 2020d; Bunderesgierung.de 2020) und alle notwendigen Server waren in Deutschland angesiedelt. Datenschutzbeauftragte und (unabhängige) Experten konnten die ausreichende datenschutzrechtliche Zuverlässigkeit der App weitgehend bestätigen (Spiegel.de 2020a; DW 2020). Andererseits gab es bereits kurz nach der Einführung der App vereinzelt Zweifel an der Datensicherheit. So haben sich bspw. bei der Einführung der App Nutzer gefragt, wieso die App bei Android-Nutzern auf den Standort zugreifen muss (Spiegel.de 2020b). Zudem wurde die Vermutung geäußert, dass die App Sicherheitslücken haben könnte, die letztlich doch zu einer Rückverfolgung persönlicher Daten führen (Zeit Online 2020d; Tagesschau.de 2020b).

**Abb. 1:**
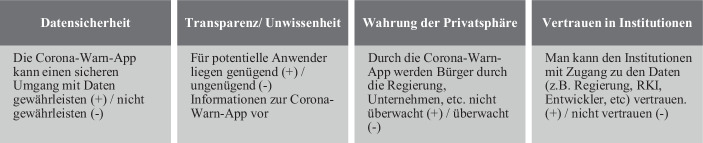
Einflussfaktoren auf die Akzeptanz der Corona-Warn-App im Hinblick auf persönliche Daten. *Anmerkungen. *Faktoren, die mit einem (+) gekennzeichnet sind, werden als potentiell positive Einflussfaktoren auf die Akzeptanz der Corona-Warn-App vermutet. Faktoren, die mit einem (−) gekennzeichnet sind, werden als potentiell negative Einflussfaktoren auf die Akzeptanz der Corona-Warn-App vermutet

Basierend auf den diskutierten Ergebnissen aus der Forschung sowie der Beobachtung der politischen und gesellschaftlichen Diskussion um die Corona-Warn-App, ergeben sich folgende Einflussfaktoren auf die Corona-Warn-App, die im Hinblick auf den Umgang mit persönlichen Daten relevant sind (Abb. 1).

### Die soziale Umwelt: Im Spannungsfeld zwischen Orientierungshilfe und Gruppenzwang

Menschen haben oft das Bedürfnis, sich bestimmten Gruppen oder der gesamten Gesellschaft zugehörig zu fühlen. Dementsprechend können sie ihr Verhalten und ihre Gewohnheiten ändern, um den Erwartungen einer definierten Gruppe zu entsprechen. Diese Übereinstimmung mit gesellschaftlichen Normen wird als *Konformität* bezeichnet (Cialdini und Goldstein 2004; Deutsch und Gerard 1955). Das Bedürfnis nach Konformität kann unterschiedlich motiviert sein: Zum einen kann die soziale Umwelt einen *informativen Einfluss* auf Individuen ausüben (Deutsch und Gerard 1955). Um bspw. mit der Unsicherheit im Hinblick auf den Umgang mit ihren persönlichen Daten besser umgehen zu können und eine Anleitung für das „richtige“ Verhalten zu finden, suchen Menschen oft nach Anhaltspunkten in ihrer sozialen Umwelt. Das können bspw. deskriptive soziale Normen sein, die das Verhalten der Mehrheit der Menschen in einer bestimmten Situation beschreiben (Cialdini 2009). Tatsächlich konnten Studien bestätigen, dass Menschen bei der Nutzung einer bestimmten Technologie eher ihre persönlichen Daten preisgeben, wenn sie dieses Verhalten bereits bei anderen beobachtet haben (Acquisti et al. 2012). Darüber hinaus kann die Gegenseitigkeit im sozialen Austausch (Reziprozität, Fehr und Schmidt 2006) entscheidend sein. Wenn Menschen andere dabei beobachten, wie sie ihre Daten für das Gemeinwohl zur Verfügung stellen, kann sie das selbst zur Offenlegung ihrer Daten motivieren. Allerdings zeigen Untersuchungen auch, dass Menschen unter Umständen absichtlich von der Gruppennorm abweichen können und ethisches Verhalten anderer sogar als moralische Lizenz für eigene Abweichungen von der Norm nutzen können (Lasarov und Hoffmann 2020). Darüber hinaus, können nicht nur die soziale Umwelt (z. B. Freunde, Familie, Gesellschaft) als Referenzpunkte dienen, sondern auch Institutionen und Regierungen (z. B. Xu et al. 2009). Die soziale Umwelt kann jedoch auch einen *normativen Einfluss* auf Menschen ausüben. Der normative Einfluss beschreibt hierbei das individuelle Bedürfnis nach Akzeptanz durch andere. Menschen wollen mit adäquatem, normgerechten Verhalten einer sozialen Sanktionierung entgehen, also der negativen Reaktion durch andere (Deutsch und Gerard 1955). Individuen würden sich also, wenn sie normativ beeinflusst sind, dem Verhalten einer Bezugsgruppe oder der Gesellschaft anpassen, um nicht unangenehm bei Nichteinhaltung bestimmter Erwartungen aufzufallen. Daraus kann auch ein sog. Konformitätsdruck entstehen, der oftmals auch als Gruppenzwang bezeichnet wird (Cialdini 2009). Im Zusammenhang mit Datenschutzbedenken könnten Menschen also Spannungen erleben, wenn sie selbst aus Angst um den Schutz ihrer Privatsphäre eine bestimmte Technologie nicht nutzen, während ihr soziales Umfeld die Technologie nutzt. Diese Spannungen vergrößern sich, wenn es sich bspw. um Technologien mit Netzwerkeffekten handelt (z. B. Kommunikationsdienste oder Technologien, die der Gesellschaft nutzen).

Ein wichtiger Aspekt in der öffentlichen Diskussion um die Corona-Warn-App bezieht sich auf den sozialen Druck und die daraus resultierende indirekt (Un)Freiwilligkeit der Nutzung. Einerseits wurde von Wissenschaftlern (Ferretti et al. 2020) und Politikern (FAZ 2020) kommuniziert, dass die App möglichst wirksam sei, wenn sie von mindestens 60–70 % der Bürger verwendet wird (Tagesspiegel.de 2020). Andererseits wurde gleichzeitig die Freiwilligkeit der Nutzung betont (Bundesregierung.de 2020). Dennoch äußerten gleich zu Beginn der Einführung der App viele Menschen Bedenken, dass unter diesem Hintergrund sozialer Druck aufgebaut werden könnte. So gab es bspw. vereinzelte Überlegungen, den Zugang zu bestimmten privaten Veranstaltungsorten an den Besitz und das Vorzeigen der App zu verknüpfen. Die Verbraucherzentralen warnten daher vor einem schleichenden Zwang zur Nutzung der Corona-Warn-App durch Arbeitgeber, Restaurants oder Behörden (Süddeutsche.de 2020). Angesichts dieser Diskussionen um den Datenschutz der App und der Gefahr der impliziten Unfreiwilligkeit, wurden kurz nach Einführung der App auch Stimmen (u. a. vom Sachverständigenrat für Verbraucherfragen, Handelsblatt.com 2020b) nach einem Begleitgesetz zur App laut, um bspw. die Zweckentfremdung durch Dritte zu verhindern oder den Einsatz der App nur auf die Zeit während der Pandemie zu beschränken.

**Abb. 2:**
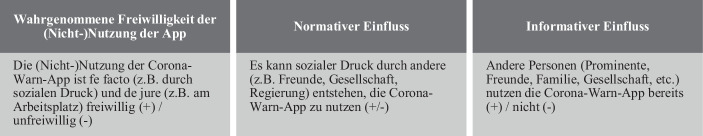
Einflussfaktoren auf die Akzeptanz der Corona-Warn-App im Hinblick auf die soziale Umwelt. *Anmerkungen. *Faktoren, die mit einem (+) gekennzeichnet sind, werden als potentiell positive Einflussfaktoren auf die Akzeptanz der Corona-Warn-App vermutet. Faktoren, die mit einem (−) gekennzeichnet sind, werden als potentiell negative Einflussfaktoren auf die Akzeptanz der Corona-Warn-App vermutet

Basierend auf den diskutierten Ergebnissen aus der Forschung sowie der Beobachtung der politischen und gesellschaftlichen Diskussion um die Corona-Warn-App, ergeben sich folgende Einflussfaktoren auf die Akzeptanz der Corona-Warn-App, die im Hinblick auf den Einfluss der sozialen Umwelt relevant sind (Abb. 2).

## Durchführung der Untersuchung und Ergebnisse

### Vorgehensweise

Als methodische Grundlage der Inhaltsanalyse dienten Kozinets’ (2016) Richtlinien für die Online-Datenerfassung und -analyse. Ähnlich zu Online-Rezensionen und Online-Kommentaren in Zeitungsartikeln, die sich bereits in einigen empirischen Studien als wertvolle Datenquelle erwiesen (z. B. Zhang et al. 2014), können auch Online-Kommentare zur Einführung der Corona-Warn-App wertvolle Erkenntnisse liefern, da es sich nicht um eine direkte Befragung von Individuen handelt und damit keine Verzerrungen aus der Befragungssituation entstehen können (Belk et al. 2006).

### Untersuchungsmaterial

Am Tag der Einführung der Corona-Warn-App (15.06.2020) wurde auf Zeit Online ein Artikel veröffentlicht, in dem die wichtigsten Informationen zur Corona-Warn-App zusammengetragen wurden (Zeit Online 2020e). Der Artikel diente also zur Information der Leserschaft und war in seiner Tonalität neutral formuliert. Es wurden u. a. der erklärte Zweck der App, die Funktionsweise, der technische Hintergrund sowie der datenschutzrechtliche Hintergrund vorgestellt. Der Artikel ist für die vorliegende Untersuchung geeignet, da die zu analysierenden Kommentare durch die neutrale Tonalität und die Breite der vorgestellten Aspekte der App nicht in einer bestimmten Richtung beeinflusst wurden. In der dazugehörigen Kommentarfunktion wurden bis zum 19.06.2020 967 Kommentare bzw. Antworten zu den Kommentaren veröffentlicht. Für die Untersuchung wurde ein Kategorienschema erstellt, das auf den Überlegungen aus dem vorangegangenen Kapitel („Aktuelle Forschung und Anwendung auf den Fall der Corona-Warn-App“) basierte. So wurden die Einflussfaktoren aus Abb. 1 entsprechend in die Kategorie „Datenschutz, Privatsphäre, Transparenz, Vertrauen“ des Kategorienschemas überführt (Tab. 1). Die Faktoren aus Abb. 2 wurden in die Kategorie „Einfluss der sozialen Umwelt“ überführt (Tab. 1). Da die Funktionalitäten einer Technologie von der jeweiligen Anwendung abhängen, wurden keine allgemeinen Einflussfaktoren hinsichtlich der Nützlichkeit für die Corona-Warn-App aus der Literatur abgeleitet. Stattdessen wurden Faktoren auf Basis einer Beobachtung der politischen und gesellschaftlichen Diskussion um die Corona-Warn-App entwickelt und daraus die Kategorie „Nutzen der Corona-Warn-App“ im Kategorienschema gebildet (Tab. 1). Zudem wurde eine Kategorie in das Kategorienschema integriert, in der die explizite Erwähnung der (Nicht‑)Installation der App kodiert wurde („Verhalten“, Tab. 1). Anhand des Kategorienschemas wurden die Kommentare kodiert. Zu jeder Unterkategorie wurde eine Erläuterung bzw. Paraphrase erstellt, die als Anleitung für die Kodierung der Artikel dienen sollte. Der Autor hat daraufhin gemeinsam mit zwei wissenschaftlichen Hilfskräften 100 Kommentare kodiert. Für Kommentare, die sich nicht in die Kategorien einordnen ließen, wurden neue Kategorien gebildet (z. B. „Zu hohe Entwicklungskosten der App“, Tab. 1). Die Kommentare wurden schließlich am 19.06.2020 von dem Autor sowie von den zwei Hilfswissenschaftlern nach dem final erstellten Kategorienschema unabhängig voneinander kodiert (Tab. 1). Abweichungen zwischen den Kodierungen wurden zwischen den Kodierenden diskutiert und ggf. angepasst. Wenn es zu keiner Übereinstimmung zwischen den Kodierern kam, wurde nach dem Mehrheitsprinzip entschieden (2:1 Stimmen). Es ergaben sich insgesamt 1092 Codes. Die Anzahl der Codes übersteigt hierbei die Anzahl der Kommentare, da in einigen Kommentaren mehrere Aspekte angesprochen wurden, die entsprechend kodiert wurden. Von den 1092 Codes wurden 425 Beiträge (39 %) von der weiteren Analyse ausgeschlossen, da sie entweder keinen inhaltlichen Bezug hatten (z. B. Beleidigungen) oder die Wiederholung einer früheren Aussage derselben Person waren.

**Tab. 1:** Ergebnisse der Untersuchung

Kategorie	Unterkategorie	Erläuterung	A	R1 (%)	R2 (%)
**Nutzen der Corona-Warn-App**			**244**	**22**	**37**
*Corona-Warn-App ist nützlich*			**84**	**8**	**13**
	Allgemein	Die App kann helfen, die Corona-Infektionen einzudämmen	69	6	10
	Gesellschaftlich	Die App kann anderen dabei helfen, nicht durch die Person infiziert zu werden	12	1	2
	Persönlich	Die App kann der Person dabei helfen, nicht durch andere infiziert zu werden	3	0	0
*Corona-Warn-App ist nicht nützlich*			**160**	**15**	**24**
	Technikprobleme	Es existieren technische Probleme, die die Nutzung der App erschweren, z. B. durch eine hohe Akkubelastung oder geringe Bluetooth-Reichweite	49	4	7
	Kritische Masse	Die App ist aufgrund mangelndem Zugang und freiwilliger Teilnahme nicht nützlich, z. B. weil viele kein Smartphone besitzen	60	5	9
	Prinzip	Das Prinzip hinter der App ist nicht sinnvoll, z. B. weil viele Personen unbemerkt infiziert sind und damit fälschlicherweise nicht markiert sind	51	5	8
**Datenschutz, Privatsphäre, Transparenz, Vertrauen**			**172**	**16**	**26**
*Datensicherheit*			**53**	**5**	**8**
	Positiv	Die Corona-Warn-App kann einen sicheren Umgang mit Daten gewährleisten	23	2	3
	Negativ	Die Corona-Warn-App kann einen sicheren Umgang mit Daten nicht gewährleisten	11	1	2
	Bedenken übertrieben	Datenschutzbedenken sind übertrieben (z. B. weil viele auch WhatsApp o. ä. nutzen). ^a^	19	2	3
*Transparenz/Unwissenheit*			**46**	**4**	**7**
	Nein	Es liegen nicht genügend Informationen zur App vor	35	3	5
	Ja	Es liegen genügend Informationen zur App vor	11	1	2
*Privatsphäre*			**31**	**3**	**5**
	Überwachung	Durch die App werden Bürger überwacht, z. B. durch die Regierung, Unternehmen, etc	15	1	2
	Keine Überwachung	Durch die App werden Bürger nicht überwacht, z. B. durch die Regierung, Unternehmen, etc	16	1	2
*Vertrauen in Institutionen*			**22**	**2**	**3**
	Vertrauen	Man kann den Institutionen mit Zugang zu den Daten nicht vertrauen, z. B. Regierung, RKI, Entwickler	22	2	3
*Begleitgesetz notwendig* ^a^			**20**	**2**	**3**
	Ja	Ein Begleitgesetz (z. B. zur Regelung der Freiwilligkeit, Datenschutz, etc.) zur App ist notwendig	15	1	2
	Nein	Ein Begleitgesetz (z. B. zur Regelung der Freiwilligkeit, Datenschutz, etc.) zur App ist nicht notwendig	5	0	1
**Einfluss der sozialen Umwelt**			**52**	**5**	**8**
*Wahrgenommene Freiwilligkeit der (Nicht‑)Nutzung der App*			**24**	**2**	**4**
	Ja	Die (Nicht‑)Nutzung der App ist faktisch und rechtlich freiwillig	15	1	2
	Nein	Die (Nicht‑)Nutzung der App ist faktisch (z. B. sozialen Druck)/rechtlich (z. B. Arbeitgeber) unfreiwillig	9	1	1
*Sozialer Druck*			**4**	**0**	**1**
	Durch Andere	Es kann sozialer Druck durch andere (z. B. Freunde, Gesellschaft) entstehen, die App zu nutzen	2	0	0
	Durch die Regierung	Es kann Druck durch die Regierung entstehen, die App zu nutzen	2	0	0
*Andere Länder als Beispiel* ^a^			**11**	**1**	**2**
	Positiv	In anderen Ländern haben ähnliche Apps die gewünschte Wirkung erzielt	3	0	0
	Negativ	In anderen Ländern haben ähnliche Apps die gewünschte Wirkung nicht erzielt	8	1	1
*Andere Menschen als Beispiel*			**13**	**1**	**2**
	Positiv	Andere Menschen (Prominente, Gesellschaft, Freunde, etc.) nutzen die App bereits	9	1	1
	Negativ	Andere Menschen (Prominente, Gesellschaft, Freunde, etc.) nutzen die App nicht	4	0	1
**Verhalten**			**79**	**7**	**12**
*Installation der App*			**79**	**7**	**12**
	Ja	App wurde/wird installiert. App sollte von anderen installiert werden	39	4	6
	Nein	App wurde/wird nicht installiert. App sollte nicht von anderen installiert werden	40	4	6
**Kein Bezug zum Nutzen, Datensicherheit, soziale Umwelt oder Nutzungsverhalten**			**545**	**50**	**18**
*Kein Bezug zur Nutzung der App*			**120**	**11**	**18**
		Erklärung technischer Details ohne Meinung/Wertung (z. B. Reichweite der Bluetooth-Verbindung)^a^	78	7	12
		Entwicklungskosten der App waren zu hoch^a^	34	3	5
		Entwicklung der App war zu spät abgeschlossen^a^	8	1	1
*Kein Bezug zum Thema Corona-Warn-App*			**425**	**39**	**–**
		Kein Bezug zum Thema (z. B. Beleidigungen)	247	23	**–**
		Antworten auf vorhergehende Kommentare (Wiederholungen)	178	16	**–**
**Gesamt**					
**Summe aller Kodierungen**			**1092**	**100**	**100**
**Summe der Kodierungen mit Bezug zum Thema Corona-Warn-App**			**667**	**61**	**100**

**Anmerkungen. ***A* Zahl der Nennungen (Absolut), *R1* Anteil der Nennungen (= A_Kategorie_/Summe aller Kodierungen), *R2* Anteil der themenrelevanten Nennungen (= A_Kategorie_/Summe der Kodierungen mit Bezug zum Thema Corona-Warn-App)

^a^ Unterkategorie wurde nach Durchsicht der ersten 100 Kommentare hinzugefügt

### Ergebnisse der Untersuchung

Zunächst kann festgestellt werden, dass die in der Vorbereitung der Studie ermittelten drei Hauptkategorien erwartungsgemäß häufig genannt wurden: Nutzen der Corona-Warn-App (37 %), Datenschutz, Privatsphäre, Transparenz, Vertrauen (26 %) sowie der Einfluss der sozialen Umwelt (8 %). Zudem haben sich 12 % der Beiträge darauf bezogen, ob die Kommentierenden die App installiert haben. Schließlich bezogen sich 12 % der Beiträge auf die Erklärung technischer Details. 5 % der Beiträge bemängelten die hohen Kosten und 1 % den späten Einführungszeitpunkt der App.

Dem Nutzen der Corona-Warn-App widmeten sich ca. ein Drittel der themenrelevanten Beiträge (37 %) von denen wiederum ca. ein Drittel den positiven Nutzen der App betonten (z. B. #602: „Wer ein MENSCH ist sollte froh sein, wenn die App vielleicht Leben rettet. Solange können Sie gerne schweigen“). 24 % der Beiträge standen der App jedoch kritisch gegenüber. Hierbei stachen besonders technische Vorbehalte hervor (z. B. #779: „Die App ist ein echter Stromfresser. Muss die Standortermittlung eingeschaltet sein?“). Ein Großteil der Beiträge kritisierte zudem, dass eine kritische Masse nicht erreicht werden konnte, weil entweder unzureichend viele Menschen ein kompatibles Smartphone nutzen (z. B. #871: „Angeblich sollten ja etwa 60 % der Bevölkerung die App herunterladen, damit sie ausreichend Erfolg hat. Jetzt haben aber überhaupt nur 60 % ein apptaugliches Handy. Was für eine Pleite.“) oder die generelle Bereitschaft zur Teilnahme zu gering sei (z. B. #265: „Die meisten werden es ignorieren, die meisten werden es nicht installieren.“). Interessanterweise wurde von einigen Kommentierenden auch der Umweltaspekt angesprochen (z. B. #866: „Aber deswegen ein neues Handy?!? Ich bin richtiggehend wütend, dass gerade umweltbewussteren Menschen, die sich nicht alle zwei Minuten ein neues Handy kaufen wollen, solche Steine in den Weg gelegt werden.“). Schließlich wurde das Prinzip der Anwendung einer Tracing-App bezweifelt (z. B. #801: „Die meisten haben keine oder milde Symptome. Woher sollen die wissen, dass sie positiv sind? Daher macht es keinen Sinn diese App zu haben“).

Beiträge zur Datensicherheit (8 %), der Transparenz/Unwissenheit (7 %), der Wahrung der Privatsphäre (5 %) sowie zum Vertrauen in die Institutionen (3 %) nahmen gemeinsam mit Diskussionsbeiträgen zur Einführung eines Begleitgesetzes (3 %) den zweitgrößten Anteil der themenrelevanten Beiträge ein (26 %). Im Hinblick auf die Datensicherheit war der Großteil der Beiträge positiv eingestellt (z. B. #5: „Hei Kyösti, viel besser kann eine App vom Datenschutz her nicht werden. Einfach mal das Whitepaper lesen oder sich den Quellcode anschauen – das ist wirklich gut umgesetzt.“) und empfand die Bedenken zum Datenschutz teilweise ungerechtfertigt (z. B. #198: „Die App geht sehr sensibel mit Daten um, da ist nichts im Vergleich zu dem was Google, Apple und Facebook zwangsinstallieren. Der Code ist offengelegt, alles sehr vorbildlich. Für die Kritik habe ich wenig Verständnis.“). Mangelndes Wissen und mangelnde Kommunikation (Transparenz/Unwissenheit) wurden in einigen Beiträgen (7 %) ebenfalls kritisiert (z. B. #751: „in der Kommunikation/Werbung/Aufklärung muss noch viel getan werden. z. B. Wie genau heißt denn die App?! Wie erkennt User überhaupt die offizielle App unter den zig anderen ähnlich benannten? Wo kann er sich ganz offiziell überhaupt über die App auf einer einfach zu findenden offiziellen Webseite informieren?“).

Überraschenderweise wurde der Einfluss der sozialen Umwelt in lediglich 8 % der Beiträge diskutiert. Hierbei bezogen sich 4 % der Beiträge auf normative Einflüsse, besonders die Orientierung an anderen Menschen und Ländern (z. B. #775: „Norwegen hat gestern die Corona-App entfernt, wegen erhebliche Datenschutzprobleme.“). Überdies haben die Kommentierenden nur zu einem geringen Anteil (4 %) die wahrgenommene Freiwilligkeit der (Nicht‑)Nutzung der App diskutiert (z. B. #15: „Es wird auch darauf ankommen, wie es am Ende mit der Freiwilligkeit in der Praxis steht. Sollte z. B. der Arbeitgeber auf Installation und Nutzung bestehen, werden sich dem viele beugen.“).

## Fazit, Limitationen und Implikationen für Forschung und Praxis

Um das volle Potenzial digitaler Technologien im Allgemeinen und der Corona-Warn-App im Speziellen ausschöpfen zu können und so das gesellschaftliche Wohlergehen nachhaltig zu verbessern, müssen psychologische Promotoren und Inhibitoren der Akzeptanz solcher Technologien identifiziert werden. Als einflussreiches Hemmnis im Umgang mit digitalen Technologien konnten in vorangegangenen Studien besonders Datenschutzbedenken identifiziert werden. Der vorliegende Beitrag hat drei Themenfelder diskutiert, die die aktuelle Forschung zum individuellen Umgang mit Datenschutzbedenken reflektieren und im Kontext der Einführung der Corona-Warn-App untersucht. Eine Inhaltsanalyse bestätigt die Relevanz der diskutierten psychologischen Faktoren im Fall der Corona-Warn-App. Auch wenn sich der vorliegende Beitrag zunächst nur explorativ möglichen Einflüssen auf die Akzeptanz der Corona-Warn-App oder ähnlicher Technologien nähert und somit keine Rückschlüsse auf Kausalitäten und Einflussstärken erlaubt, lassen sich erste Hinweise auf mögliche Maßnahmen zur Erhöhung der Akzeptanz vermuten: So kann eine möglichst transparente Informationspolitik eine wirksame Maßnahme sein, da 5 % der untersuchten Beiträge sich darauf bezogen, dass nicht genügend Informationen über die Corona-Warn-App zur Verfügung standen oder diese nicht die potentiellen Anwender der App erreichten. Gerade in Zeiten von sogenannten *Fake News* ist eine flächendeckende Aufklärung notwendig, die sich nicht nur auf die Vermittlung von Sachinformationen stützt, sondern ebenfalls auf Gerüchte reagiert, die im Internet verbreitet werden. Weiterhin kann der vorliegende Beitrag eine Grundlage schaffen, um bisherige Forschung zum Datenschutz und Anwenderverhalten theoretisch zu erweitern. So können anhand der Corona-Warn-App neue, kontextspezifische Konstrukte entwickelt werden, die das Nutzungsverhalten von Individuen beeinflussen. So werden mit der Nutzung der App bspw. nicht nur unmittelbare individuelle Bedürfnisse befriedigt, wie sonst üblich, sondern auch gesellschaftliche Bedürfnisse erfüllt. Ein weiterer wichtiger Faktor könnte in diesem Zusammenhang sein, wie sehr sozialer Druck Reaktanz bei potentiellen Anwendern auslösen kann.

Eine Limitation der Studie ist, dass die Leserschaft von Zeit Online tendenziell weiblich, jünger und höher gebildet als der Bevölkerungsdurchschnitt ist (meedia.de 2013). Daher kann die Gewichtung der Faktoren in der Gesamtbevölkerung etwas abweichen. Eine weitere Inhaltsanalyse auf dem Facebook-Auftritt von BILD News mit 89 Online-Kommentaren zu einem Artikel, der ebenso neutral in der Tonalität war („Installation und Nutzung: Die neue Corona-Warn-App Schritt für Schritt erklärt“), ergab eine Verteilung der kodierten Beiträge, die ähnlich zu der Verteilung auf der Kommentarseite von Zeit Online war, sich allerdings deutlich stärker auf die Diskussion um den Datenschutz konzentrierte: 59 % der inhaltsbezogenen Einträge waren auf den Datenschutz und die Datensicherheit bezogen. 11 % bezogen sich auf den Nutzen der App. Lediglich 5 % der Beiträge bezogen sich auf Aspekte der sozialen Konformität und 25 % darauf, ob die App installiert oder nicht installiert wurde. Da Studien auf zwei verschiedenen Plattformen mit unterschiedlichem Klientel durchgeführt wurden und eine hohe Anzahl an Kommentaren (>1000) ausgewertet wurde, zeichnen die ermittelten psychologischen Einflüsse auf die Akzeptanz Corona-Warn-App ein sehr umfangreiches Bild. Allerdings sollten, um die Repräsentativität der Ergebnisse zu erhöhen und die psychologischen Faktoren ggf. um weitere Faktoren zu ergänzen, andere Online-Plattformen in weiteren Studien untersucht werden (bspw. Twitter). Zudem sollten auch Meinungen erfasst werden, die nicht online kommuniziert wurden, um auch in dieser Hinsicht Verzerrungen durch die Art der Erhebung zu vermeiden (z. B. sind ältere Menschen tendenziell unterrepräsentiert auf Online-Plattformen). Zukünftige Forschung sollte außerdem die hier identifizierten Faktoren mit großzahligen und repräsentativen empirischen Befragungen untersuchen, um Kausalitäten und Effektstärken zu ermitteln.

## Footnotes

1 Der wesentliche Baustein einer Tracing-Warn-App ist die Nutzung der Bluetooth-Technologie. Hiermit werden lediglich die relevanten „digitalen Handschläge“ erfasst, also ob überhaupt ein relevanter Kontakt stattgefunden hat. Nicht erfasst wird, wo diese Kontakte stattgefunden haben. Ein Standort-Tracking, wie es etwa über GPS oder das Mobilfunknetz möglich wäre, findet durch die Corona-Warn-App also nicht statt (Verbraucherzentrale.de 2020).
